# Re-irradiation of anaplastic meningioma: higher dose and concomitant Bevacizumab may improve progression-free survival

**DOI:** 10.1186/s13014-024-02486-7

**Published:** 2024-10-02

**Authors:** Ory Haisraely, Alicia Taliansky, Maayan Sivan, Yaacov Lawerence

**Affiliations:** Sheba Medical Center, Tel Aviv Medical School, Tel Aviv University, Tel Aviv, Israel

**Keywords:** Anaplastic meningioma, Re-irradiation, Dose escalation, Bevacizumab

## Abstract

**Introduction:**

Anaplastic meningiomas, categorized as WHO grade 3 tumors, are rare and highly aggressive, accounting for 1-2% of all meningioma cases. Despite aggressive treatment, including surgery and Radiation, they exhibit a high recurrence rate and poor survival outcomes. The aggressive histopathological features emphasize the urgent need for effective management strategies.

**Methods:**

A retrospective multi-institutional analysis was conducted on patients with recurrent anaplastic meningioma who underwent re-irradiation between 2017 and 2023. Clinical, dosimetric, and outcome data were collected and analyzed, focusing on local control, progression free survival and treatment-related adverse events.

**Results:**

Thirty-four cases were analyzed, with a median follow-up 11 months after re-irradiation. Progression-free survival at 12 months was 61.9%, with higher doses correlating with better outcomes. Concomitant Bevacizumab improves progression-free survival and reduces the risk of radiation necrosis. CDKN2A homozygote deletion correlated with a higher risk of local failure. Symptomatic radiation necrosis occurred in 20.5% of cases, but its incidence was lower with concomitant Bevacizumab treatment.

**Conclusion:**

Re-irradiation presents a viable option for recurrent anaplastic meningioma despite the associated risk of radiation necrosis. Higher doses with concomitant Bevacizumab improve clinical outcomes and reduce toxicity. Individualized treatment approaches are necessary, emphasizing the importance of further research to refine management strategies for this challenging disease.

## Introduction

Anaplastic meningiomas, classified as(World Health Organization) WHO grade 3 tumors, represent an uncommon yet highly aggressive subset of meningiomas, constituting only 1-2% of all meningioma cases [1]. Distinguished by their particularly malignant clinical course, anaplastic meningiomas pose a significant therapeutic challenge, marked by a propensity for local recurrence and an overall poor survival outcome. Despite advancements in neuro-oncology, these tumors exhibit a 3-year progression rate of 59.2%, even following gross total resection and adjuvant Radiation, with an associated overall survival of 78.6% [2].

The histopathological hallmark of anaplastic meningioma is high mitotic activity, cellular atypia, and frequent necrosis, with 2021 WHO incorporating genomics and molecular data into this classification [3, 4]. These aggressive features underscore the urgent need for effective management strategies, especially in recurrent diseases, where treatment guidelines are notably scarce. Literature on the toxicity associated with re-irradiation in this specific clinical setting is conspicuously limited, accentuating the necessity for a comprehensive evaluation of outcomes related to local control, overall survival, and treatment-related adverse events.

In this multi-institutional analysis, we aim to address the existing knowledge gap by systematically examining re-irradiation outcomes in cases of recurrent anaplastic meningioma. Our focus extends beyond survival metrics to encompass factors influencing local control and the potential toxicities associated with re-irradiation.

## Methods

Following institutional review board approval, a retrospective review was conducted on patients undergoing re-irradiation for recurrent or progressive anaplastic meningioma at three major medical centers between 2017 and 2023. Clinical, dosimetric, and outcome data were collected and analyzed. The biological effective dose was calculated using an alpha-beta ratio of 10. Kaplan-Meier and Cox regression analyses were performed to describe hazard ratios for local control and survival, utilizing SPSS software (IBM, Chicago, USA version 29). All pathological data were re-analyzed by central evaluation to ensure that both pathologies represented anaplastic meningioma as defined by the WHO 2021 classification. All pathological specimens were also analyzed using fluorescent in-situ hybridization to evaluate 1P del and CDKN2A deletion status (defined as deletion if more than 50%). Local progression was defiend by The Response Assessment in Neuro-Oncology (RANO) 2.0 criteria [5].

## Results

Thirty-four cases of recurrent anaplastic meningioma that received a second course of Radiation were analyzed. All re-radiation treatments were in field recurrence. The median time from the first radiation course was 11 months (7–25 months). The average age was 57.2, with 55.8% being females. Mitotic rate was the diagnostic criterion for 70% of patients, with 10 exhibiting CDKN2A homozygote deletions. For the initial presentation, 88% underwent gross tumor resection (Simpson 1–3) and received Radiation with a median dose of 60 Gy (54–60 Gy), translating to a biologically effective dose (using an alpha/beta ratio of 10) of 72 Gy (64.8–72). In the recurrent setting, 73% underwent a second surgery (Simpson > 3), and all patients received a second course of Radiation with a median dose of 48.8 Gy (range 31.2–56). In 47% of patients, the 2nd (radiation therapy) RT dose was higher than 50 Gy biological effective e dose (BED). In all radiation planning, the clinical target volume (CTV) was defined as the disease identified on a T1 + G MRI with a planning target volume(PTV) between 0.3 cm and 0.5 cm isotropic expansion.

Bevacizumab (Bev) was administered concomitantly in 35.3% of cases.

The median follow-up from the second Radiation was 13 months (range 5–28), and progression-free survival at 12 months was 61.9% (Table 1).

### Impact of dose

**Fig. 1:**
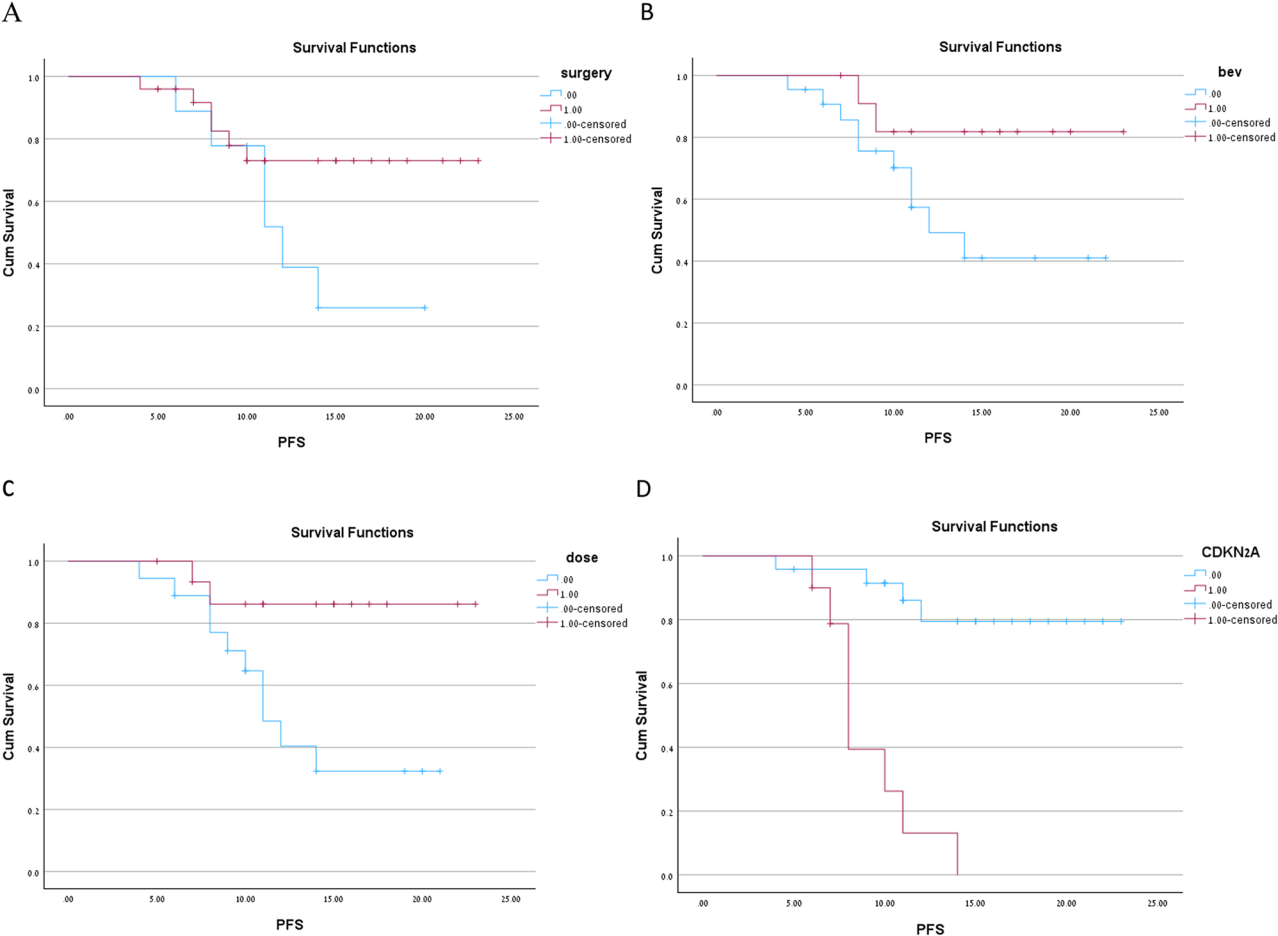
Kaplan Meir curve for Progressio nfree survival (PFS) for four variabels. **A**-Dose (1-above 50Gy, 0- below 50Gy). **B**-2nd surgery (1-yes, 0-no). **C**-CDKN2A status (1 -deletion, 0-wt). **D**-Concomittent Bevacizumab (1-yes, 0-no)

The average dose was higher for those who achieved local control than those who did not (51.4 Gy vs. 42.6 Gy, *p* < 0.001). Among patients who achieved local control, 68.1% received a dose higher than 50 Gy; in comparison, only one patient who received the 2nd RT dose above 50 Gy had a local failure and received a dose lower than 50 Gy (*p* = 0.012). There was no significant difference in other variables between those who received more than 50 Gy and those who did not. Patients who received an RT dose above 50 Gy (BED) were less likely to progress with an HR = 0.3 {CI95% 0.045-0.8}. higher dose above 50gy improve progression free survival (*p* = 0.03) (Fig  1).

### Impact of surgery

second surgery did not impact Progreession-free survival outcomes in our cohort with HR 0.36 (0.2–1.27, *p* = 0.109). (Fig  1). All second surgeries were classified as Simpson grading 1–3. Age (median 51 years VS 69 years) and tumor location (parietal more prevalent in those who underwent second surgery and frontal in those who did not) were the only variables differing between those who underwent second surgery and those who did not.

### Impact of bevacizumab

Most (66%) of the patients receiving bevacizumab concomitant with 2nd RT received it because they experienced neurological symptoms related to mass effect secondary to edema. The remaining four patients received it due to physician preference. A statistical trend for improving progression-free survival did not reach statistical significance with using bevacizumab.

All univariable analyses and Kaplan Mair are shown in Table 2; Fig. 1.

**Table 1:** Patient’s characteristics

*n*	34
Age	57.2y (37–74)
Location of primary tumor	
Frontal Parietal Temporal Occipital Base of skull	18 10 4 1 1
Type of resection 1st surgery (Simpson)	
1–2 3–4 5	12 20 2
Reason for WHO 3	
Histopathology criteria CDKN2A deletion Mitotic > 20	16 10 24
1p loss (more than 50%)	55.8%
Karnofsky status	80 (70–100)
Total dose deliver 1st	60 Gy (54–60)
2nd surgery (yes)	25 (73%)
Total dose deliver 2nd	35 Gy (25-40.05)
Number of fractions	10 (5–15)
BED 2nd RT (α/β = 10) Median, range	48.8 (31.2–56)
Bed > 50 Gy	16 (47%)
concomitant Bevacizumab (yes)%	12 (35.3%)
PTV 2^ND^ RT	35 cc (11.5-212.4 cc)
Median follow up (range)	13 (5–28 months)

**Table 2:** Univariable analysis on using cox model regression

Variable	HR (CI95%)	*P*
RT > 50gy	0.3 (0.045-0.8)	0.03
2^ND^ surgery (yes)	0.39 (0.12–1.2)	0.109
Bevacizumab concomittemt (yes)	0.27 (0.06–1.27)	0.1
CDKN2A homozygous deletion (yes)	11 (3.1–38.4)	< 0.001

Multivariable Cox regression model

**Fig. 2:**
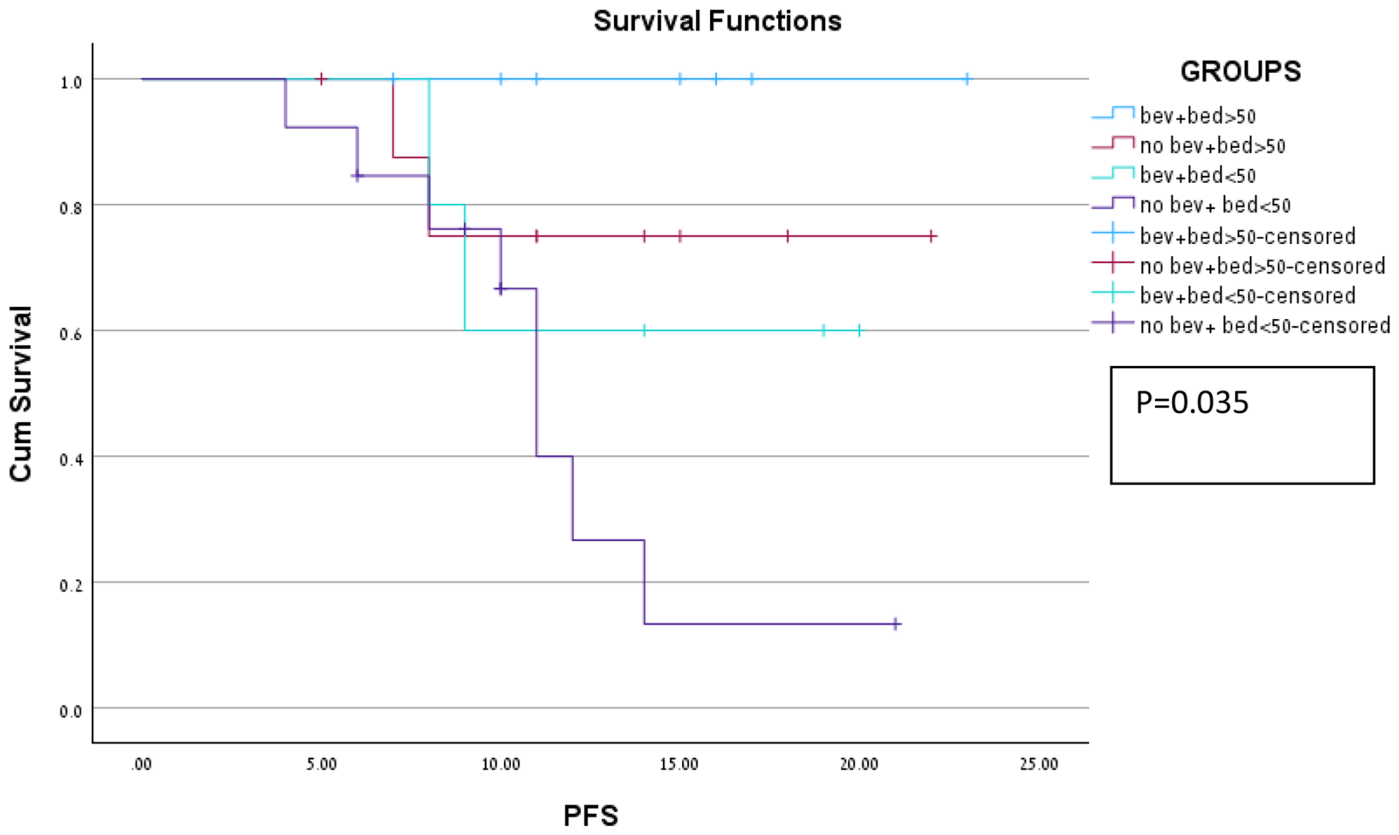
Kaplan Meir curve for PFS among 4 groups. Blue-dose >50gy+concomittent Bevacizumab. Red-dose>50gy alone. Light green-dose<50gy + concomittent bevacizumab. Purple-dose>50Gy

When incorporating both Bevacziuamb use and dose distribution, we created four different groups. Among the groups, patients who received a dose above 50 Gy concomitant with Bevacizumab had no local progression at one year in our cohort. Patients who received a dose above 50 Gy and did not receive Bevacizumab had 25% 1-year local Patients who received Bevacizumab with a dose lower than 50 Gy had a 40% local progression. Finally, patients who did not receive a dose above 50 Gy and did not receive Bevacizumab had 53% local progression at one year. There was a significant change in progression-free survival ( PFS) between groups (*p* = 0.035). Kaplan Mair curves for all four groups are shown in Fig. 2.

### Impact of histopathological data and genetic factors

Ten patients had CDK2NA homozygote deletion. Having CDKN2A homozygote deletion increased the hazard ratio for PFS by 11.1 (3.1–38.4), *p* < 0.001. There was no influence on 1P status or mitotic activity.

### Toxicity report

There was a 20.5% incidence of symptomatic radiation necrosis (RN). Most cases (71.4%) presented with headache. In addition, 1 with seizure, and 1 with confusion and disinhibition. All patients with symptomatic RN improved with dexamethasone treatment. A combined dose for both 1st and 2nd radiation treatment of more than 120 Gy (BED) increased the odds for RN (HR-2.4 {1.3–4.1).

There were no cases of RN in those who received Bevacizumab concomitant with RT in the recurrent setting.

## Discussion

### Reoperation

Surgical intervention alleviates the mass effect, relieves associated neurological symptoms, decreases the risk of recurrence, and provides for diagnosis and molecular characterization.5 All of these factors are important when discussing the advantages of reoperation. However, despite plentiful data on the morbidity and mortality associated with initial meningioma resection, information on re-resection in recurrent cases still needs to be determined. A recent analysis found that 48% of the reoperation cases experienced at least one complication in their postoperative care. Pre-radiation tumor location and the experience of the neurosurgical team are essential variables in this context [6, 7].

### Genomic factors

In our cohort, ten patients had CDKN2NA homozygote deletion (more than 50%). With the recent 2021 update of the WHO Classification of Central Nervous System Tumors, homozygous deletions of *CDKN2A/B* are sufficient to classify meningiomas as CNS WHO grade 3 tumors regardless of histological grading [3]. Meningiomas harboring homozygous deletions of *CDKN2A/B* are characterized by high recurrence rates independent of WHO grade, DNA methylation class, sex, age, and tumor location [4]. Additionally, heterozygous loss, mutations, and promoter methylation of *CDKN2A* were strongly associated with recurrent meningiomas and a high Ki-67 index [8]. Physiologically, the proteins encoded by *CDKN2A/B* halt the cell cycle; consequently, homozygous loss leads to dysregulated cell cycle progression and uncontrolled proliferation [8]. In our cohort, a strong relationship was observed between disease recurrence after 2nd RT and CDKN2a Del. Anaplastic meningioma with CDKN2A loss has a poor prognosis, even in re-radiation.

### Dose regiment and bevacizumab treatment

The optimal RT approach for grade 3 meningioma in the recurrent setting remains controversial regarding clinical target volume (CTV) margins and dose prescription. Regarding dosage regimens for in-field recurrence, data on hypofractionation treatment and stereotactic radiosurgery exist, primarily relating to atypical rather than anaplastic meningioma.

The dose-response relationship is more established in anaplastic meningioma, as evidenced by publications in the adjuvant setting. Pontoriero et al. demonstrated that in 16 patients who received 72.5 Gy EQD2, treated with a combination of IMRT and radiosurgery, a 3-year PFS of 75% in sub-totally resected or recurrent grade 2 meningioma [9]. Lee et al., In a cohort of 21 patients who received IMRT to a median GTV dose of 66 Gy (range, 63–69 Gy), reported 3- and 5-year PFS rates of 88.4% and 73.5%. With a mixed photon/proton technique, [10] Chan et al. escalated doses to 68.4 to 72 Gy and found that 5 of the six patients with grade 2 or 3 meningioma achieved long-term local control at a mean follow-up of 145 months [11]. Moreover, most recently, Zhen et al. concluded that the dose-escalation cohort (≥ 66 Gy equivalent dose) improves local control and PFS (HR-0.42) [10].

Dose escalation above 50 Gy (BED), for example, 40 Gy in 10 fractions or 32.5 Gy in 5 fractions, appears crucial for improving progression-free survival at the cost of a high RN rate.

Concomitant RT with BEV in anaplastic glioma is a rare practice. This practice is an extrapolation from managing recurrent high-grade gliomas, particularly the 1205 RTOG trial, which showed improved progression-regression-free survival with the combination of RT and BEV versus BEV alone [12].

Our data showed improvement in ln progression-free survival with the use of BEV. The group with the best oncology results was the group for which both a higher dose was given and a Bevacizumab concomitant treatment with no progression after one year of follow-up.

In addition, an interesting observation was the decreased incidence of RNs using BEV.

With Second RT, one of the significant side effects is RN, which has been reported to rise to 25% for cumulative EQD2 > 130 Gy using an α/β ratio of 2 Gy for a normal healthy brain. The use of BEV has been shown to improve radiation necrosis. A recent meta-analysis reported that radiographic responses were recorded in 84.7% of patients, and clinical improvement was observed in 91% [13].

One limitation of Bevacizumab is its side effects and the chance of increasing surgical complications [14]. In our cohort, most of the patients who received concomitant Bevacizumab did not undergo a second surgery, so perhaps increasing the dose with concomitant Bevacizumab can be more relevant for patients for whom second surgery is not possible.

### Strengths and limitations

Our study has several limitations. The rarity of this presentation limited Our sample size. Additionally, the retrospective nature of this study identified only patients with complete clinical and dosimetric data, potentially introducing record bias. The small sample size limited our ability to perform multivariable analysis. Nevertheless, the strength of our study lies in collecting data from multiple hospitals, with thorough reviews of surgical reports and plans. All histopathological data underwent review, and genomic analyses were performed to classify anaplastic meningioma according to the WHO 2021 classification.

## Conclusions

*Anaplastic meningioma* is a rare disease characterized by a poor prognosis and lacks established guidelines for recurrent disease management. Re-irradiation presents a viable option, albeit with a 20% risk of symptomatic RN, particularly if the combined dose exceeds 120 Gy. CDKN2A status is pivotal for achieving progression-free survival in the recurrent setting. Concomitant BEV administration improves progression-free survival and reduces the risk of radiation necrosis. Further research is warranted to refine strategies for managing recurrent anaplastic meningioma, emphasizing individualized treatment approaches.

## Data Availability

No datasets were generated or analysed during the current study.

## Abbreviations

WHO: Worls Health Organization
RT: Radiation Therapy
PFS: Progression Free Survival
Bev: Bevacizumab
CTV: Clinical Target Volume
PTV: Planning Target Volume
